# Construction of a machine learning-based artificial neural network for discriminating PANoptosis related subgroups to predict prognosis in low-grade gliomas

**DOI:** 10.1038/s41598-022-26389-3

**Published:** 2022-12-21

**Authors:** GuanFei Chen, ZhongMing He, Wenbo Jiang, LuLu Li, Bo Luo, XiaoYu Wang, XiaoLi Zheng

**Affiliations:** 1 School of Basic Medical Sciences, Southwest Medical University, Luzhou, 646000 China; 2 Department of Neurosurgery, Qingdao Municipal Hospital, Qingdao University, Qingdao, 266011 China

**Keywords:** Computational biology and bioinformatics, Data mining, Genome informatics, Machine learning

## Abstract

The poor prognosis of gliomas necessitates the search for biomarkers for predicting clinical outcomes. Recent studies have shown that PANoptosis play an important role in tumor progression. However, the role of PANoptosis in in gliomas has not been fully clarified.Low-grade gliomas (LGGs) from TCGA and CGGA database were classified into two PANoptosis patterns based on the expression of PANoptosis related genes (PRGs) using consensus clustering method, followed which the differentially expressed genes (DEGs) between two PANoptosis patterns were defined as PANoptosis related gene signature. Subsequently, LGGs were separated into two PANoptosis related gene clusters with distinct prognosis based on PANoptosis related gene signature. Univariate and multivariate cox regression analysis confirmed the prognostic values of PANoptosis related gene cluster, based on which a nomogram model was constructed to predict the prognosis in LGGs. ESTIMATE algorithm, MCP counter and CIBERSORT algorithm were utilized to explore the distinct characteristics of tumor microenvironment (TME) between two PANoptosis related gene clusters. Furthermore, an artificial neural network (ANN) model based on machine learning methods was developed to discriminate distinct PANoptosis related gene clusters. Two external datasets were used to verify the performance of the ANN model. The Human Protein Atlas website and western blotting were utilized to confirm the expression of the featured genes involved the ANN model. We developed a machine learning based ANN model for discriminating PANoptosis related subgroups with drawing implications in predicting prognosis in gliomas.

## Introduction

Low-grade gliomas (LGGs) comprising grade II and III gliomas represent a group of primary tumors affecting cells of the central nervous system. Grade II and III gliomas are common in young adults compared with high-grade gliomas (grade IV, glioblastoma multiforme, GBM)^1^. In 2021, the WHO (World Health Organization) updated the classification method for gliomas by combining histological diagnosis with molecular variations such as *IDH* and H3 G34 mutation status and co-deletion of the short arm of chromosome 1 and the long arm of chromosome 19 (1p/19q codeletion)^2^. Previous studies demonstrated that glioma patients with mutant *IDH* exhibited a more favorable response to current therapies including radiation and chemotherapy, implying that a correlation exist between molecular alterations and prognosis^3^. Given its high heterogeneity, glioma patients show diverse clinical outcomes even when they have the same diagnosis^4^. Although the survival of LGG patients tend to be longer, its median overall survival ranges from 5.6 to 13.3 years, indicating that the prognosis of LGG patients is highly variable^5,6^. Therefore, it important to identify biomarkers for predicting the prognosis of cancer.

As a significant hallmark of cancer, resistance to cell death plays an important role in tumorigenesis and tumor progression^7^. Apoptosis serves as a classical programed cell death (PCD) mechanism in the past decades. Current treatment strategies aiming to induce apoptosis in cancer cells exert less satisfactory therapeutic response^8^. Identification of an alternative, novel PCD pathway may be urgently needed. Pyroptosis, which is activated by gasdermin (GSDM) protein family^9^, and necroptosis, which is mediated by RIPK3-dependent MLKL oligomerization^10^, have been drawing more and more attention in recent years. However, an accumulating number of studies have demonstrated that pyroptosis, apoptosis and necroptosis are extensively cross-linked. PANoptosis, which shares common key features with pyroptosis, apoptosis and/or necroptosis, is determined as an inflammatory PCD pathway and cannot be simply accounted for by any of these three identified PCD pathways alone^11^. It is well documented that PANoptosis which is induced by specific factors such as inflammatory triggers and cytokines can be regulated by the PANoptosome complex^12,13^. Scholars have revealed the inhibitory effect of PANoptosis on tumor growth in diverse cancer lineages, shedding more light on the investigation of biomarkers and therapeutic targets for patients^14^. However, the specific roles of PANoptosis in glioma prognosis remain to be defined. To date, there is few literatures aiming to characterize PANoptosis related patterns or identify PANoptosis related gene signature in gliomas.

Neural networks are well-known and have been widely used in previous bioinformatical models due to their outstanding performance^15,16^. In this study, we identified two distinct PANoptosis related molecular patterns based on the expression profiles of PANoptosis related genes (PRGs), followed which PANoptosis related gene signature and two PANoptosis related gene clusters were determined, which was closely associated with the prognosis of LGG patients. Subsequently, machine learning algorithms including least absolute shrinkage and selection operator (LASSO) logistic regression and support vector machine-recursive feature elimination (SVM-RFE) were utilized to identify featured genes for characterizing two PANoptosis related gene clusters. Considering the difficulties when few genes were selected for the separation between two PANoptosis related gene clusters, we employed artificial neural network (ANN) to perform non-linear modeling to achieve a higher accuracy.

## Results

### Determination of PANoptosis related molecular patterns

**Figure 1:**
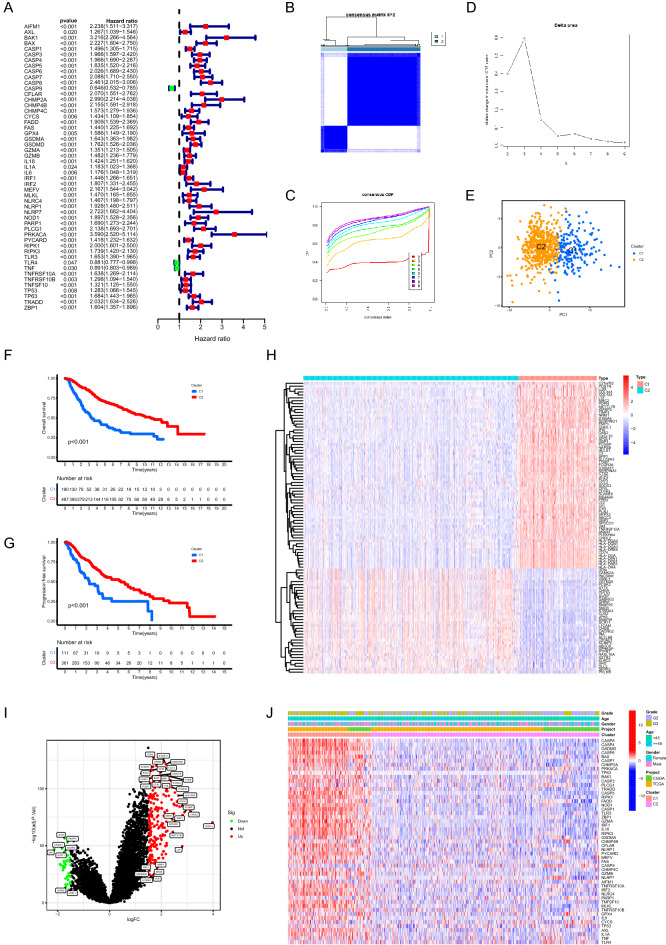
Determination of PANoptosis related molecular patterns. (**A**) Forest plot showing PRGs with prognostic values. (**B–D**) Determination of two PANoptosis related clusters via consensus clustering analysis based on the expression profiles of prognostic PRGs. (**E**) PCA of the classification of LGG samples based on prognostic PRGs. (**F,G**) Kaplan–Meier analysis showing the comparisons of overall survival (**F**) and progression free survival (**G**) between two clusters. (**H**) The expression patterns of DEGs between two clusters. (**I**) Volcano plot showing the DEGs between two clusters. (**J**) The differential expression patterns of PRGs between two clusters. *PRGs* PANoptosis related genes, *PCA* principal component analysis, *LGG* low-grade gliomas, *DEGs* differentially expressed genes.

Firstly, 51 PRGs with prognostic values were screened out via univariate cox regression analysis (Fig. 1A), based on which two distinct PANoptosis related molecular patterns were determined in LGG samples by consensus clustering method. As shown in the consensus matrix heatmap, we found extremely higher consensus scores between samples in the same cluster and lower scores between samples in different clusters when k = 2 (Fig. 1B). Moreover, no evident increase was detected with respect to the values of the area under the CDF curve when k = 2 (relative change = 0.4, Fig. 1C,D). The detailed results of the consensus clustering analysis were shown in Supplementary Fig. 1A (k ranging from 3 to 9). The results of PCA demonstrated that LGG samples could be appropriately distinguished based on the expression profiles of prognostic PRGs (Fig. 1E). The prognosis of C1 was worse compared to C2, in which the overall survival and progression free survival of C1 were significantly shorter (Fig. 1F,G). The DEGs between two clusters were visualized in Fig. 1H,I. In addition, we found differential expression patterns of PRGs between two clusters which were contextually defined as two PANoptosis related molecular patterns (Fig. 1J). The comparisons of the expression levels of prognostic PRGs between two PANoptosis related molecular patterns were demonstrated in Supplementary Fig. 1B.

### Identification of PANoptosis related gene clusters

**Figure 2:**
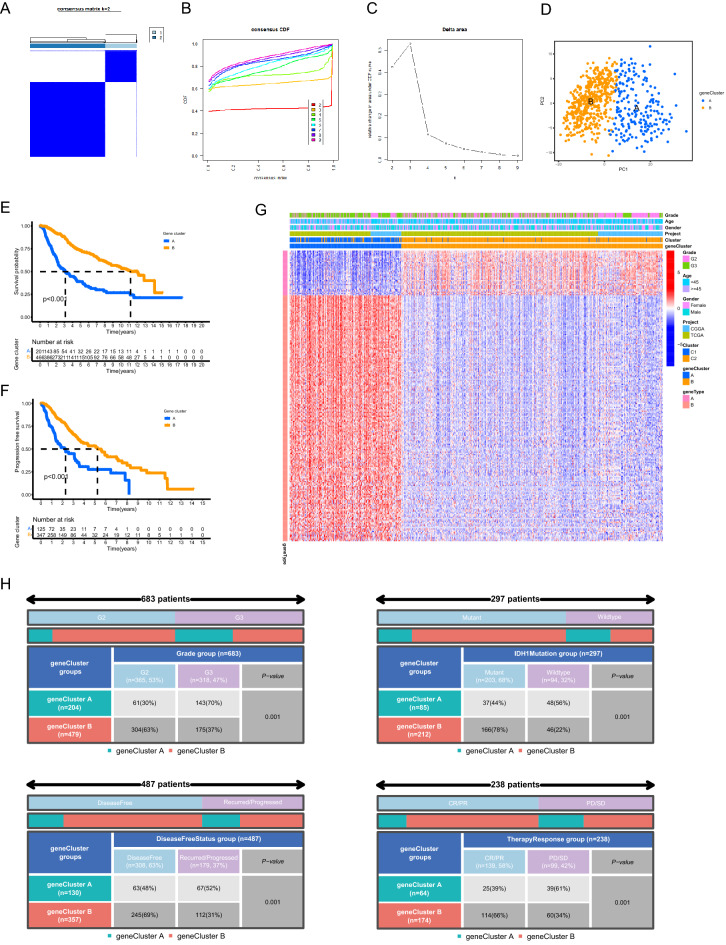
Identification of PANoptosis related gene clusters. (**A–C**) Determination of two PANoptosis related gene clusters based on the expression profiles of PANoptosis related gene signature. (**D**) PCA of the classification of LGG samples based on the expression profiles of PANoptosis related gene signature. (**E,F**) Kaplan–Meier analysis showing the comparisons of overall survival. (**E**) and progression free survival (**F**) between two gene clusters. (**G**) The differential expression patterns of PANoptosis related gene signature between two gene clusters. (**H**) Comparisons of multiple clinicopathological characteristics between two gene clusters. *PCA* principal component analysis, *LGG* low-grade gliomas, *CR/PR* complete response/partial response, *PD/SD* progressed disease/stable disease.

The prognostic DEGs between two PANoptosis related molecular patterns were screened out and subsequently defined as PANoptosis related gene signature, based on which two distinct PANoptosis related gene clusters were identified through similar clustering method (Fig. 2A–C). The detailed results of the consensus clustering analysis were shown in Supplementary Fig. 2A (k ranging from 3 to 9). PCA confirmed the subgroup assignment based on the expression profiles of PANoptosis related gene signature (Fig. 2D). The corresponding clinicopathological information for LGG samples from TCGA and CGGA databases was listed in Supplementary Table 1 and Supplementary Table 2, respectively. Kaplan–Meier analysis indicated that the prognosis of gene cluster A was substantially worse than those of gene cluster B (Fig. 2E,F). The differential expression patterns of PANoptosis related gene signature between two gene clusters was presented in Fig. 2G, in which the expression level of gene type A positively correlated with gene cluster A while the expression level of gene type B positively correlated with gene cluster B. Furthermore, we found that multiple clinicopathological characteristics significantly differed between two PANoptosis related gene clusters. The histopathological grade, the proportion of *IDH1* with wild type and the proportion of recurred or progressed tumors of gene cluster A were higher than those of gene cluster B. Moreover, gene cluster A was less likely to respond to current treatment compared to gene cluster B (Fig. 2H). All these findings indicated poor prognosis for patients of gene cluster A.

### Functional enrichment analysis between PANoptosis related gene clusters

**Figure 3:**
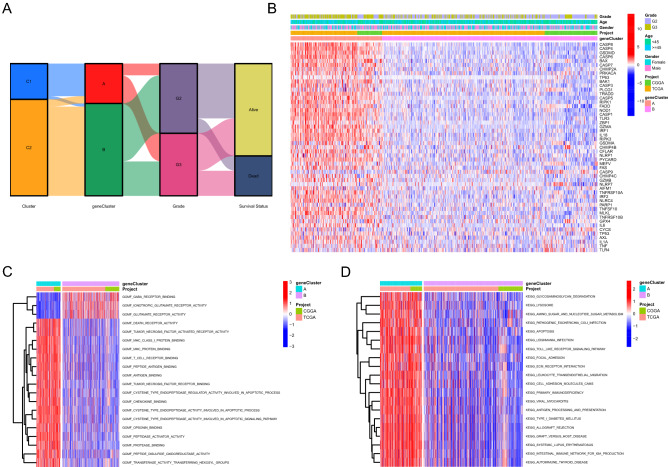
Functional enrichment analysis between PANoptosis related gene clusters. (**A**) Alluvial diagram showing the correlation across PANoptosis related molecular patterns, gene clusters, histological grade and survival status. (**B**) The differential expression patterns of PRGs between two gene clusters. (**C**) The differential enrichment of molecular functions between two gene clusters. (**D**) The differential enrichment of pathways between two gene clusters. *PRGs* PANoptosis related genes, *GO* gene ontology, *KEGG* Kyoto encyclopedia of genes and genomes.

The correlation across PANoptosis related molecular patterns, gene clusters, histological grade and survival status was shown in Fig. 3A, in which PANoptosis related molecular patterns C1 almost overlapped with gene clusters A. Consistent with the above results, most of PRGs were highly expressed in PANoptosis related gene cluster A (Fig. 3B). The comparisons of the expression levels of prognostic PRGs between two PANoptosis related gene clusters were demonstrated in Supplementary Fig. 2B. Immune related molecular functions including tumor necrosis factor activated receptor activity, T cell receptor binding and MHC protein binding were significantly enriched in gene cluster A (Fig. 3C). Tumorigenesis and tumor progression related KEGG pathways including ECM receptor interaction, focal adhesion and apoptosis were significantly enriched in gene cluster A (Fig. 3D).

### Evaluation of the performance of PANoptosis related gene clusters in discriminating prognosis

**Figure 4:**
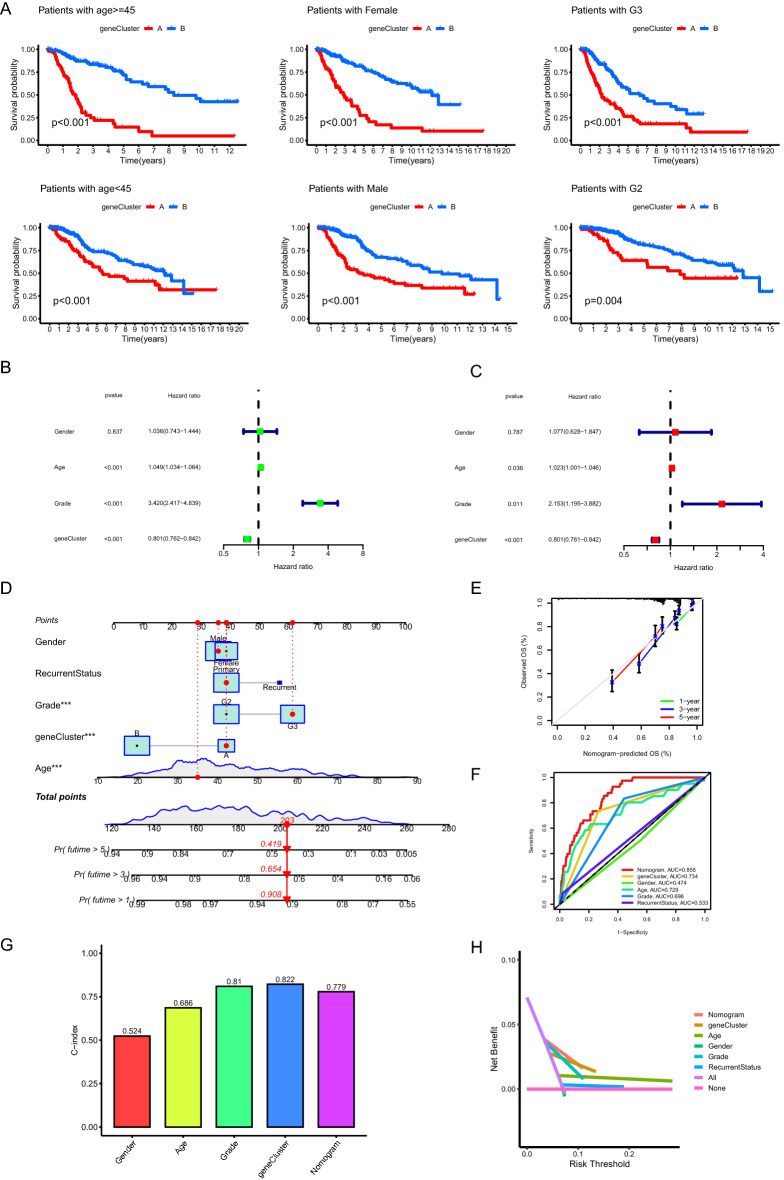
Evaluation of the performance of PANoptosis related gene clusters in discriminating prognosis. (**A**) Comparisons of the overall survival between two gene clusters with distinct clinicopathological features. (**B,C**) Univariate (**B**) and multivariate (**C**) cox regression analysis indicated the roles of gene cluster in discriminating prognosis. (**D**) Construction of nomogram model by combining gene cluster and multiple clinicopathological factors. (**E**) Calibration curves for the nomogram model. (**F**) ROC curves showing the performance of the nomogram model for predicting 1-year overall survival. (**G**) C-index of the nomogram model and gene cluster for predicting prognosis. (**H**) DCA presenting the performance of the nomogram model and gene cluster in discriminating prognosis. *ROC* receiver operating characteristic, *C-index* consistency index, *DCA* decision curve analysis.

Patients with distinct clinicopathological features were further grouped into two PANoptosis related gene clusters, in which the overall survival of gene cluster A was still significantly shorter than those of gene cluster B indicating the powerful performance of gene cluster in discriminating prognosis in LGGs (Fig. 4A). Univariate cox analysis demonstrated that the PANoptosis related gene cluster was closely associated with the overall survival of LGG patients (Fig. 4B, p < 0.001) and multivariate cox analysis confirmed that the gene cluster served as an independent prognostic factor (Fig. 4C, p < 0.001). For clinical practice, a nomogram model combining PANoptosis related gene cluster and multiple clinicopathological factors was constructed to predict 1, 3 and 5-year overall survival of LGG patients (Fig. 4D). As depicted in Fig. 4E, the predicted 1, 3 and 5-year overall survival by the nomogram model simulated the observed overall survival. In addition, the AUC value of the nomogram model for predicting 1-year survival was 0.855 which was higher than those of other clinicopathological factors (Fig. 4F). The C-index for PANoptosis related gene cluster and nomogram model were 0.822 and 0.799, respectively (Fig. 4G). The result of DCA for the nomogram model further verified its powerful performance in discriminating prognosis (Fig. 4H).

### Exploration of the distinct TME between PANoptosis related gene clusters

**Figure 5:**
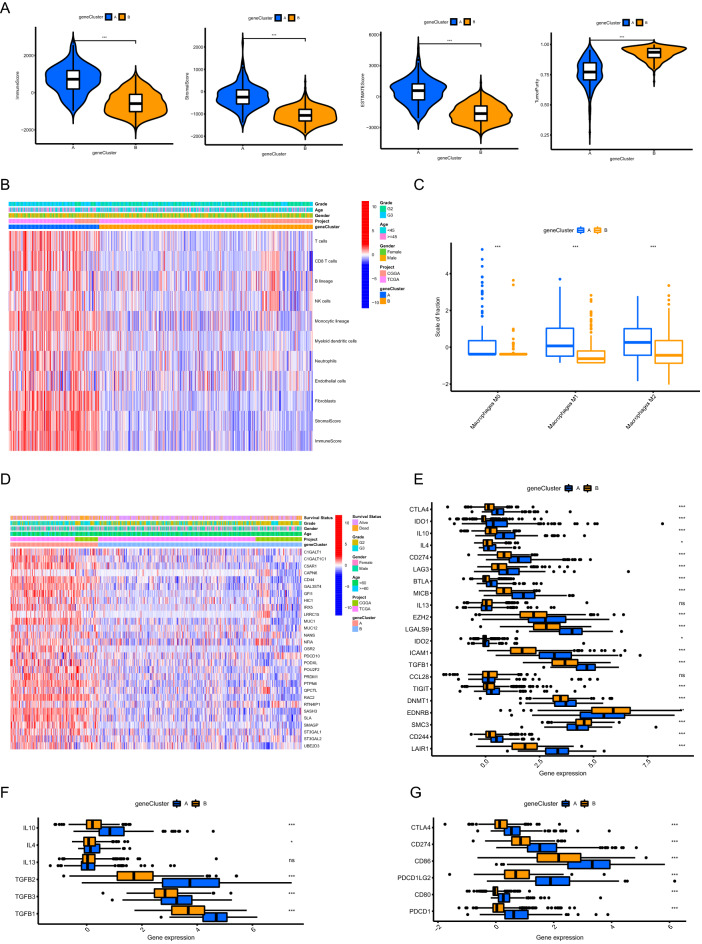
Exploration of the distinct TME between PANoptosis related gene clusters. (**A**) Comparisons of immune scores, stromal scores, ESTIMATE scores and tumor purity. (**B**) The differential patterns of the abundance of infiltrating cells in TME calculated by MCP counter. (**C**) Comparisons of the abundance of macrophages infiltrated in TME calculated by CIBERSORT algorithm. (**D**) The differential expression patterns of the genes involved in negative regulation of ADCP. (**E**) Comparisons of the expression levels of the genes involved in negative regulation of Cancer-Immunity Cycle. (**F**) Comparisons of the expression levels of immune suppressive cytokines. (**G**) Comparisons of the expression levels of common immune checkpoints. *TME* tumor microenvironment, *ADCP* antibody-dependent cellular phagocytosis, *p < 0.05, **p < 0.01, ***p < 0.001.

Based on ESTIMATE algorithm, we found that the immune, stromal and ESTIMATE scores of PANoptosis related gene cluster A were significantly higher than those of gene cluster B (Fig. 5A, p < 0.001), indicating more non-tumor compositions existing in TME. Consistent with the above results, the tumor purity of gene cluster A was lower compared to gene cluster B (p < 0.001). As calculated by MCP counter, we found that more immune and stromal cells infiltrated in the TME of gene cluster A, especially T cells, monocytic lineage, myeloid dendritic cells and fibroblasts (Fig. 5B, Supplementary Fig. 3A, p < 0.001). CIBERSORT algorithm was utilized to calculate the abundance of macrophages in TME. Interestingly, we found that more macrophages infiltrated in the TME of gene cluster A, including macrophages M0, macrophages M1 and macrophages M2 (Fig. 5C, p < 0.001). Considering that macrophages play an important role in antibody-dependent cellular phagocytosis (ADCP) of cancer cells^17^, we analyzed the expression of genes involved in the negative regulation of ADCP. As shown in Fig. 5D and Supplementary Fig. 3B, most of the identified genes were highly expressed in PANoptosis related gene cluster A (p < 0.001), suggesting that ADCP was inhibited in gene cluster A. With respect to the genes involved in negative regulation of Cancer-Immunity Cycle^18,19^, we found that most of these genes were highly expressed in gene cluster A, indicating gene cluster A has low activities of antitumor immune processes (Fig. 5E). Moreover, most of the immune suppressive cytokines (IL-10, IL-4, TGF-β) induced by macrophages and regulatory T cells were also significantly overexpressed in gene cluster A (Fig. 5F)^20,21^. All the common immune checkpoints were upregulated in gene cluster A (Fig. 5G, p < 0.001). These findings suggested that PANoptosis related gene cluster A presented a suppressive anti-tumor immunity phenotype which might contribute to poor prognosis.

### Construction of ANN for discriminating PANoptosis related gene clusters

**Figure 6:**
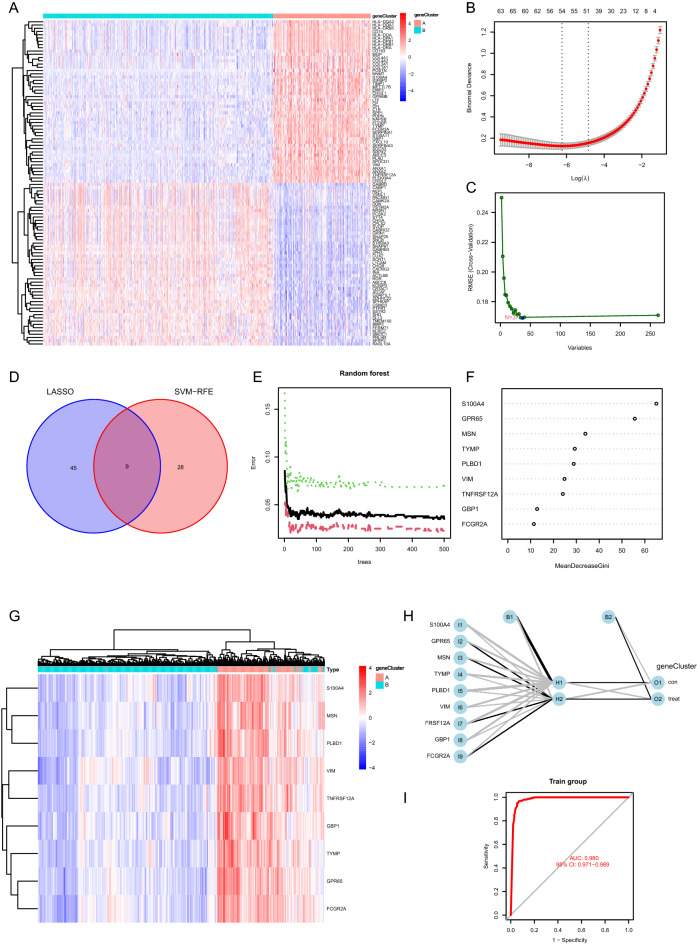
Construction of ANN for discriminating PANoptosis related gene clusters. (**A**) The expression patterns of DEGs between two gene clusters. (**B**) Determination of the optimal number of featured genes by LASSO logistic regression. (**C**) Determination of the optimal number of featured genes by using SVM − RFE algorithm. (**D**) Venn plot showing the overlapped featured genes obtained by the above methods. (**E**) The optimal number of the random forest trees was determined when the cross-validation error presented minimal. The red dots represented the samples in gene cluster A, the green dots represented the samples in gene cluster B, and the black dots represented all the samples. (**F**) The feature importance for the nine featured genes. (**G**) Unsupervised clustering for glioma samples based on the expression of nine featured genes. (**H**) The ANN for discriminating two PANoptosis related gene clusters based on the expression of nine featured genes. (**I**) ROC curve showing the efficacy of the ANN for discriminating two PANoptosis related gene clusters. *ANN* artificial neural network, *DEG* differentially expressed gene, *LASSO* least absolute shrinkage and selection operator, *SVM-RFE* support vector machine-recursive feature elimination, *ROC* receiver operating characteristic.

The differential expression patterns of DEGs between two PANoptosis related gene clusters were depicted in Fig. 6A. Firstly, LASSO logistic regression machine learning method was utilized to identify the feature genes for discriminating two PANoptosis related gene clusters, in which 54 featured genes was determined when the lambda value was minimal (Fig. 6B). Subsequently, SVM-RFE machine learning algorithm was performed to further determine the featured genes, in which 37 featured genes was identified when RMSE was minimal (Fig. 6C). We obtained nine overlapped genes via the above two methods, including S100A4, GPR65, MSN, TYMP, PLBD1, VIM, TNFRSF12A, GBP1, FCGR2A (Fig. 6D). ROC curves showing the efficacy of each featured gene for discriminating two PANoptosis related gene clusters were demonstrated in Supplementary Fig. 4, in which all the AUC values were higher than 0.920. Moreover, random forest was used to further screen out featured genes based on the expression profiles of the above nine featured genes, in which 42 trees were determined when the cross-validation error presented minimal (Fig. 6E). Based on the determination of the optimal number of forest trees, the feature importance for each gene was calculated, in which we found that the feature importance for each gene was higher than 10 (Fig. 6F). Unsupervised clustering for LGG samples was conducted based on the expression of nine featured genes. We found that samples in the same PANoptosis related gene cluster tended to be grouped into one cluster, indicating that LGG samples could be well distinguished through the expression of the featured genes (Fig. 6G). The ANN model for discriminating two PANoptosis related gene clusters was constructed based on the expression of nine featured genes (Fig. 6H). The formulas in the ANN model for producing the values of O1 and O2 were as follows:

$$F1 \, Hi=Bi+{\sum }_{k=1}^{9}COEFik*Ik,$$

in which H*i* (H1, H2) represented the value of the *i-*th neurons in the hidden layer, B*i* (B1, B2) represented the basic value when calculating the *i-*th value in the hidden layer, I*k* (I1, … ,I9) represented the input value of the *k*-th featured gene, *COEFik* represented the coefficient when calculating the value of the *i*-th neuron in the hidden layer by using the *k*-th featured gene.

$$F2 \,Oi=Bi+{\sum }_{k=1}^{2}COEFik*Hk,$$

in which O*i* (O1, O2) represented the *i-*th value in the output layer, B*i* (B1, B2) represented the basic value when calculating the *i-*th value in the output layer, H*k* (H1, H2) represented the value of the *k*-th neuron in hidden layer, *COEFik* represented the coefficient when calculating the *i*-th value in the output layer by using the value of *k*-th neuron in the hidden layer. The corresponding coefficients in the formulas were shown in Table 1. As depicted in Fig. 6I, the AUC value for the ROC curve which represented the efficacy of the ANN for discriminating two PANoptosis related gene clusters was 0.980, indicating the outstanding performance of the model.

**Table 1:** Coefficients in the formulas of ANN.

	H1	H2
Basic value	10.6634674	0.908588424
I1	− 1.8262535	0.012442761
I2	− 1.2341838	− 0.051344195
I3	− 0.9984977	− 0.0217154
I4	− 1.0985669	− 0.02459666
I5	− 2.6356201	0.012115227
I6	− 0.6953330	− 0.002427534
I7	− 2.5898937	− 0.002118653
I8	− 0.6585406	0.009224131
I9	− 0.8451296	0.032968312

	O1	O2
Basic value	− 0.7252137	0.9194955
H1	0.4688068	− 0.4764947
H2	1.7467213	− 0.6105463

### Validation of the ANN model in independent external datasets

**Figure 7:**
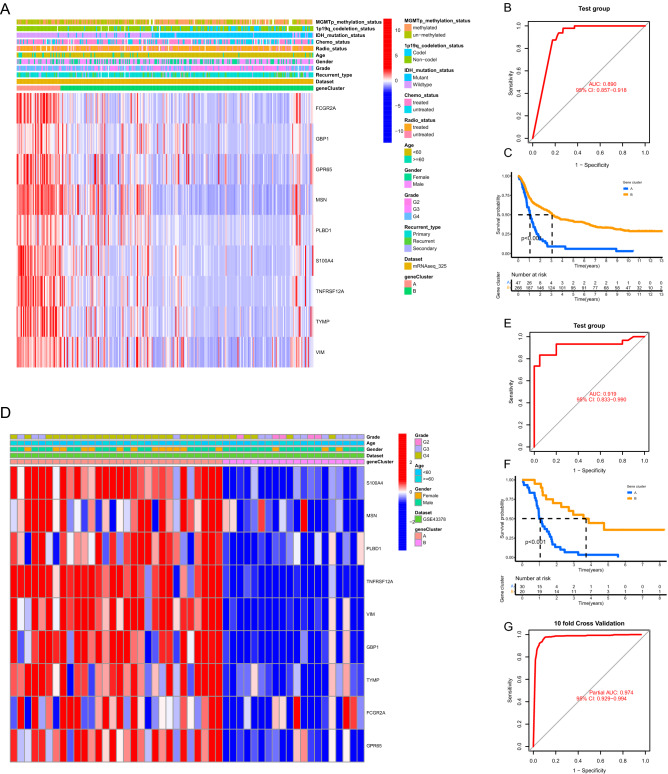
Validation of the ANN model in independent external datasets. (**A**) The differential expression patterns of the featured genes between two PANoptosis related gene clusters in validation dataset from CGGA database (dataset ID: mRNAseq_325). (**B**) ROC curve showing the efficacy of the ANN model for discriminating two PANoptosis related gene clusters in the validation dataset from CGGA database. (**C**) Kaplan–Meier analysis showing the comparisons of overall survival between two gene clusters in the validation dataset from CGGA database. (**D–F**) Similar results obtained in the validation dataset from GEO database (GSE43378). *ANN* artificial neural network, *ROC* receiver operating characteristic. (**G**) ROC curve showing the efficacy of the in the validation dataset from the ten-fold cross-validation data.

Based on the expression profiles of PANoptosis related gene signature, glioma samples in the two validation cohorts were classified into two gene clusters and the detailed results of the consensus clustering analysis were depicted in Supplementary Fig. 5A,B. As shown in Fig. 7A, all the nine featured genes were highly expressed in PANoptosis related gene cluster A, which was consistent with the results acquired in the training cohort. The ROC curve of the ANN model for discriminating PANoptosis related gene clusters was demonstrated in Fig. 7B. In addition, the overall survival of gene cluster A was significantly shorter than those of gene cluster B (Fig. 7C). Similar results were obtained in the validation dataset from GEO database (GSE43378) (Fig. 7D–F). All these findings confirmed that our ANN model could discriminate distinct PANoptosis related gene clusters in gliomas. In the result of ten-fold cross-validation method, Since there is no crossover between the training set and the test set in each cross-validation process, over-learning of the model can be prevented. The model accuracy of each cycle in the cross-validation process is shown in Supplementary Table 3. The ROC curve of the ten-fold cross-validation method in Fig. 7G.Furthermore, the performance of the ANN model was evaluated by using multiple metrics, including specificity, sensitivity, accuracy and AUC values. As shown in Table 2, the specificity, sensitivity, accuracy and AUC values were 0.956, 0.950, 0.954 and 0.98 in the training cohort (train), 0.754, 0.937, 0.781 and 0.89 in the validation cohort from CGGA database (mRNAseq_325), 1.000, 0.666, 0.80 and 0.92 in the validation cohort from GEO database (GSE43378), 0.935, 0.888, 0.922 and 0.97 in the trainCV, respectively.

**Table 2:** Performance results of ANN in three independent datasets.

Dataset	TN	FN	TP	FP	Specificity	Sensitivity	Accuracy	AUC
Train	460	10	194	21	0.9563410	0.9509804	0.9547445	0.98
mRNAseq_325	209	3	45	68	0.754512	0.9375000	0.781538	0.89
GSE43378	20	10	20	0	1	0.6666667	0.8000000	0.92
trainCV	117	3.75	7.8	45.3	0.9358631	0.8887498	0.9226744	0.97

### Validation of the featured genes involved in the ANN model at protein level

**Figure 8:**
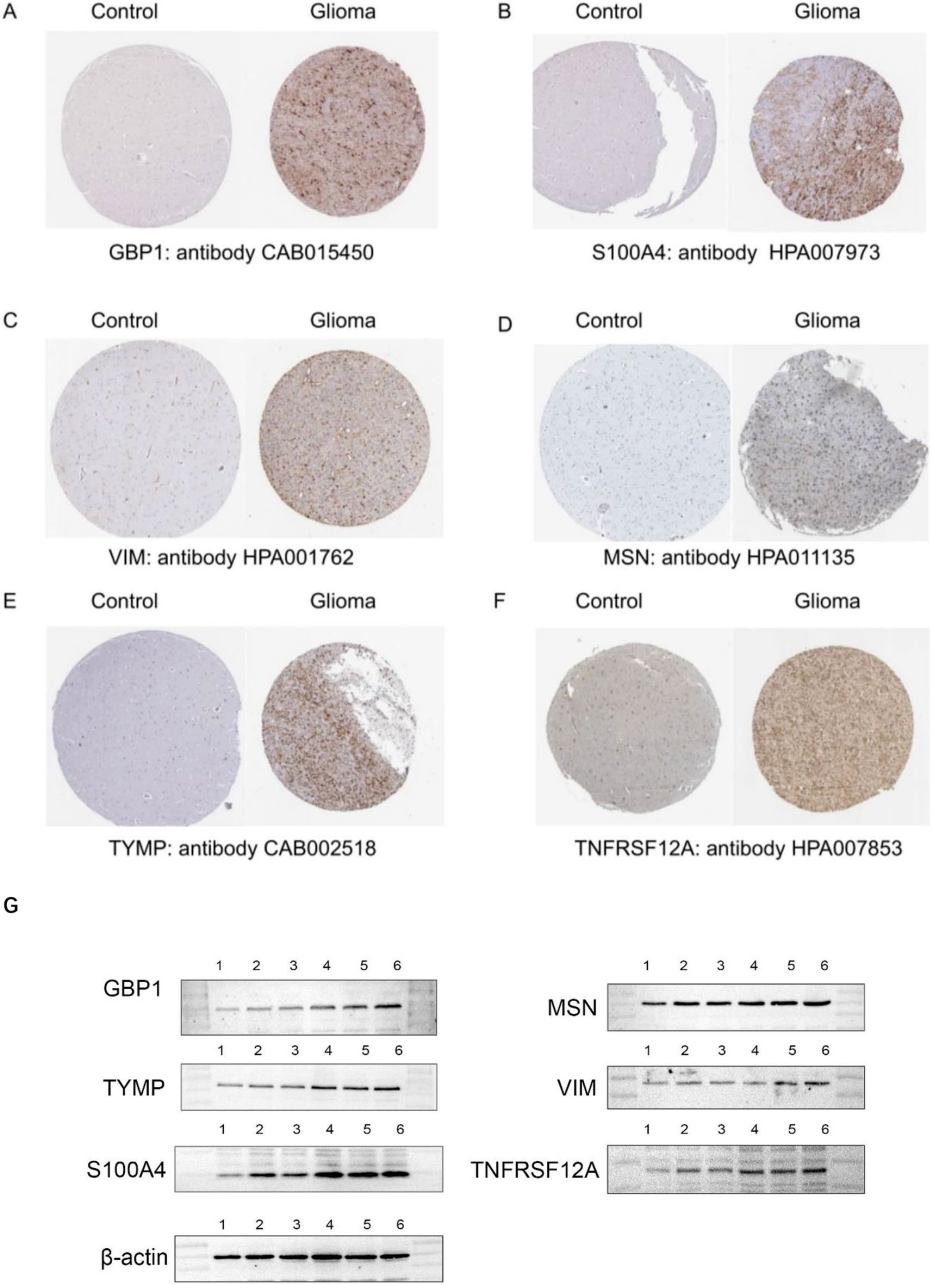
Validation of the featured genes involved in the ANN model at protein level. (**A–F**) The differential expression patterns of the featured genes between normal brain tissues and glioma tissues which were identified in immunohistochemistry staining on the Human Protein Atlas website. (**G**) Identification of the featured genes by western blotting, in which lane 1 represented normal brain tissues, lane 2 and 3 represented grade II glioma tissues, lane 4 and 5 represented grade III glioma tissues, lane 6 represented grade IV glioma tissues. Control: normal brain tissue. Original blots are presented in Supplementary material.

Six genes including *GBP1, S100A4, TYMP, TNFRSF12A, VIM* and *MSN* were randomly selected from the nine featured genes involved in the ANN model. We found differential expression patterns of the above genes between normal and glioma tissues in immunohistochemistry staining on the Human Protein Atlas website (Fig. 8A–F). Western blotting confirmed the high expression levels of the six genes in glioma tissues at protein level (Fig. 8G).

## Discussion

As an inflammatory PCD pathway, PANoptosis which is characterized by extensively activation of pyroptosis (GSMDs), apoptosis (CASP8/3/7) and necroptosis (pMLKL) related molecules, has been widely implicated in various settings including microbial infection, inflammatory diseases and cancers^22^. For example, excessive production of cytokines induced by inflammatory cell death (PANoptosis), can lead to a life-threatening condition, which is defined as cytokine storm and is involved in a large number of diseases, including the ongoing COVID-19 pandemic^23^. On the other hand, PANoptosis may play a positive role with respect to cancer due to its capacity to kill cancer cells^14^. However, the correlation between the alteration of expression patterns of PRGs and the prognostication in gliomas is rarely reported before. In our study, two PANoptosis related molecular patterns with distinct prognosis were identified based on comprehensive analysis of the expression profiles of PRGs in LGGs. Based on the DEGs between two PANoptosis related molecular patterns, which were subsequently defined as PANoprosis related gene signature, LGG samples were classified into two PANoptosis related gene clusters. We found that PANoptosis related gene cluster A showed worse prognosis due to positive correlation with tumor grade and resistance to current therapeutics. In addition, PANoptosis related gene cluster A exhibited suppressive anti-tumor immunity compared to gene cluster B. Machine learning algorithms were utilized to select featured genes to characterize PANoptosis related gene clusters, followed which ANN was employed to perform non-linear modeling for discriminating distinct PANoptosis related gene clusters.

In recent years, despite the fact that several gene signatures have been established for predicting the prognosis of glioma patients^24–26^, none of them was associated with PANoptosis. Moreover, samples were simply separated into low or high-risk groups with distinct prognosis based on the specific gene signature, lacking sufficient biological interpretations. We determined two PANoptosis related gene clusters with distinct prognosis and proved that the PANoptosis related gene cluster served as an independent prognostic factor. For promotion of clinical practice, a nomogram model consisting of PANoptosis related gene cluster and multiple clinicopathological characteristics was constructed, with high AUC values (0.855 and 0.865 at 1 and 2 years) for predicting the prognosis in gliomas. The results of C-index and DCA confirmed the outstanding performance of the nomogram model. Interestingly, most of PRGs were highly expressed in PANoptosis related gene cluster A compared to gene cluster B, including critical molecules usually activated in PANoptosis pathway such as CASP8/3/7, GSDMD and RIPK3/1. In addition, glioma patients were classified into two PANoptosis related molecular patterns (C1, C2) based on the expression profiles of PRGs by using consensus clustering analysis. Similarly, most of PRGs were highly expressed in C1 compared to C2. As shown in Fig. 2G and Fig. 3A, PANoptosis related molecular pattern C1 almost overlapped with gene cluster A, which was in accordance with our findings. These results revealed that the two gene clusters represented distinct PANoptosis patterns, in which glioma cells were prone to PANoptosis in gene cluster A. In contrary to our findings, Malireddi et al. demonstrated that PANoptosis inhibited tumor growth in diverse cancer lineages^14^. In fact, tumor cells in the core or inner regions of many forms of solid tumors may suffer from oxygen and glucose deprivation due to ischemic conditions, which induces necroptotic cell death^27^. Moreover, emerging data have indicated that necroptosis could promote cancer progression, implying that necroptosis acts as a double-edged sword in the development of cancer^28^. Sugimoto et al. suggested that high proportion of tumor necrosis predicted poor prognosis in surgically resected high-grade tumors^29^. Apoptosis is reported to be very common in many types of cancer, particularly high-grade forms^30^. Thus, this raises the possibility that, in the context of oxygen and glucose deprivation, tumor cells with high aggressiveness are susceptible to PANoptosis, resulting in the alteration of the expression patterns of PRGs. Furthermore, high intratumoral heterogeneity, which could be explored by single-cell analysis at the resolution of cells, might account for this anomalous result to some extent^31^. Further exploration is needed to confirm the activation of PANoptosis in subgroups of gliomas, in which more cellular and molecular biological experiments in vitro and in vivo may provide more evidence in the in the future.

Further analysis probed into the distinct TME between two PANoptosis related gene clusters. It has been well documented that cancer associated fibroblasts (CAFs) inhibit the functions of immune cell in TME and promote tumor progression by secretion of various cytokines and/or metabolic products. Moreover, CAFs inhibit the infiltration of immune cells in TME by reshaping the extracellular matrix^32,33^. The majority of non-neoplastic cells in TME, tumor associated macrophages (TAMs) have been reported to play an important role in tumor progression^34^, in which they are reshaped by cytokines such as IL-4 and IL-10 to impede anti-tumor immunity^21^. Consistent with the previous studies, we found more non-tumor compositions in PANoptosis related gene cluster A, especially fibroblasts and macrophages, which was further confirmed by MCP counter and CIBERSORT algorithm. Cytokines (IL-4, IL-10 and TGF-β) involved in immunosuppressive process and common immune checkpoints (PD-1 and CTLA-4) were significantly upregulated in gene cluster A, indicating an immunosuppressive pattern in gene cluster A. In addition, critical molecules participating in the negative regulation of ADCP were highly expressed in gene cluster A, suggesting the effect of ADCP was inhibited in gene cluster A. All these findings appealed implications in the correlation between TME and PANoptosis related gene cluster, directing the application of immunotherapy for gliomas.

Considering the failures in targeting apoptosis in cancer therapy in some forms of cancers^8^, induction of PANoptosis in cancer cells may be probably preferred for the exploration of promising therapeutics in cancer cells bearing defects in classic PCD pathways. Actually, the widely-accepted oncolytic viruses, such as vaccinia virus and vesicular stomatitis virus, have been reported to potentially induce PANoptosis^22^. In our study, the identification of glioma-specific PANoptosis related gene signature and the corresponding molecular mechanisms provided the underlying therapeutic targets with respect to PANoptosis in gliomas.

In the modern era, artificial intelligence (AI) has been broadly used for construction of prediction model due to its powerful capacity in extracting and representing data^35^. In present study, machine learning based ANN, which served as a sub-concept of AI method, was constructed to increase our ability to accurately discriminate PANoptosis related gene clusters with distinct prognosis.

There were still some limitations in our study. Firstly, despite that the classification of gliomas based on PANoptosis related gene signature and the robust performance of ANN model were verified in two independent external datasets including gliomas with grade II, III and IV, our research focused on the exploration of PANoptosis in patients with LGGs, in which the training cohort (merged data) mainly contained LGGs. Moreover, the expression of featured genes in ANN model were further confirmed in gliomas with grade II, III and IV. Secondly, only the alterations of gene expression were taken into consideration in our study. More evidence including DNA methylation and genomic mutation signature might be needed in the further research. Thirdly, although the tumor heterogeneity across individuals was involved in the analysis of RAN-seq data and western blotting, the intratumoral heterogeneity in gliomas was not fully addressed.

Overall, our study provided a PANoptosis related gene signature, based on which we determined two PANoptosis related gene clusters and two PANoptosis related molecular patterns with distinct prognosis. According to the latest classification for primary tumors in central nervous system, published in 2021, the importance of molecular diagnostics has been further underscored, such as molecular mutation status and DNA methylome profiling^2^. As described in our study, many PANoptosis related genes were determined to be associated with prognosis of glioma patients. The prognostic PANoptosis related genes may be taken into consideration for the classification of gliomas to precisely predict the prognosis in the future. For example, S100A4 in our study, was highly expressed in patients with poor prognosis and served as an independent predictor. Gliomas with high expression level of S100A4 may be more likely to be classified into more higher grade in the next edition of WHO classification.

In our study, based on the expression of PANoptosis related gene signature in gliomas, samples were classified into distinct PANoptosis related gene clusters, which appealed implications in predicting clinical outcomes in gliomas. Furthermore, an ANN model based on machine learning methods was developed to characterize and discriminate distinct PANoptosis related gene clusters.

## Materials and methods

### Data acquisition

A dataset containing 508 LGG samples with the corresponding RNA sequencing (RNA-seq) data were downloaded from the TCGA database (The Cancer Genome Atlas, http://cancergenome.nih.gov/). The annotation file, Genome Reference Consortium Human Build 38 (GRCh38), which was acquired from the Ensembl website (http://asia.ensembl.org/), was utilized to annotate the RNA-seq data. The microarray data (dataset ID: mRNA-array_301) composed of 159 LGG samples were obtained from CGGA database (Chinese Glioma Genome Atlas, http://cgga.org.cn/index.jsp)^36,37^. The corresponding clinical information for LGG patients in the two datasets was also downloaded from the above websites. In our study, 78 PRGs were identified through extensive scanning of pyroptosis, apoptosis, necroptosis and PANoptosis-related literatures^9–11,22,38–42^. Bioinformatic analysis and visualization of the data was performed using the R software (version 4.1.1).

### Determination of PANoptosis related molecular patterns

The high throughput RNA-seq data obtained from TCGA database were transformed into transcripts per million (TPM) values and then transferred into the log2 scale. Afterwards, the microarray data obtained from CGGA database (dataset ID: mRNA-array_301) were merged with the RNA-seq data from TCGA database. The data were then normalized and the batch effect was corrected before further analysis. R packages including limma and sva packages were used for the above analyzes^43,44^.

Firstly, 51 PRGs with prognostic significance were identified in the univariate cox regression analysis with the survival R package. PRGs with p < 0.05 were considered to be statistically significant. Thereafter, distinct PANoptosis-related molecular patterns were determined based on the expression profiles of 51 prognostic PRGs in the merged data through consensus clustering method using the ConsensusClusterPlus R package^45^. Subsequently, clustering analysis based on the Partitioning Around Medoid (PAM) algorithm which was conducted using k-means machine learning algorithm. A total of 50 repetitions were carried out in the consensus clustering analysis to determine the stability of our classification and 80% of the LGG samples were used in each iteration. The optimal number for subgroup assignment of LGG samples was comprehensively determined with the consensus matrix heatmap and the relative change values of the area under the cumulative distribution function (CDF) curves. Principal component analysis (PCA) was performed to assess the results of subtype assignment in relation to the expression profiles of the 51 prognostic PRGs in LGGs.

### Identification of PANoptosis related gene clusters

DEGs (differentially expressed genes) between distinct PANoptosis related molecular patterns were determined by |log2 FC (fold change) |> 1.5 and FDR (false discovery rate) adjusted p values < 0.05 via limma package in R software^46^. Univariate cox regression analysis was applied to select the prognosis related DEGs which were defined as PANoptosis related gene signature in LGGs. The consensus clustering analysis was utilized to identify distinct PANoptosis related gene clusters based on the expression profiles of the PANoptosis related gene signature.

### Functional enrichment analysis

GSVA (gene set variation analysis) was implemented for the function annotation of distinct subgroups by using the GSVA package in R software^47^. The differentially enriched molecular mechanisms including Gene Ontology (GO) molecular function terms and Kyoto Encyclopedia of Genes and Genomes (KEGG) pathways between subgroups were identified by using limma package in R^44^, in which |log_2_FC|> 0.1 and FDR adjusted p values < 0.05 were considered significantly enriched between subgroups. The reference files including “c5.go.mf.v7.4.symbols” and “c2.cp.kegg.v7.4.symbols” were downloaded from GSEA database (https://www.gsea-msigdb.org/).

### Evaluation of the performance of PANoptosis related classification in predicting prognosis

The prognostic values of PANoptosis related gene clusters were verified by univariate and multivariate cox regression analysis and visualized in forest plots. For clinical practice, a nomogram model combining PANoptosis related gene clusters and multiple clinicopathological features was constructed to efficiently predict the prognosis of LGG patients by using rms and regplot R packages. The calibration curves, ROC (receiver operating characteristic) curves, DCA (decision curve analysis) and C-index (consistency index) were presented to evaluate the performance of the nomogram model in predicting prognosis in LGGs.

### Exploration of tumor microenvironment (TME)

TME in LGGs was quantified via ESTIMATE algorithm (Estimation of STromal and Immune cells in MAlignant Tumour tissues using Expression data), by which immune, stromal, ESTIMATE score (positively reflecting nontumor components) and tumor purity were calculated based on the estimate R package^48^. The relative abundance of essential immune and stromal cells in the TME was quantified via MCP counter^49^. CIBERSORT, a deconvolution algorithm based on linear support vector regression, was employed to further evaluate the abundance of immune infiltrating cells in TME based on the gene expression profiles of LGG samples^50^.

### Identification of the featured genes for PANoptosis related gene clusters

We screened out DEGs between distinct PANoptosis related gene clusters by |log2 FC |> 1.5 and FDR < 0.05 via limma R package^46^, based on which two machine learning algorithms were adopted to select the key genes for discriminating PANoptosis related gene clusters, including least absolute shrinkage and selection operator (LASSO) logistic regression^51^ and support vector machine-recursive feature elimination (SVM-RFE)^52^. LASSO serves as a special instance of the penalized least squares regression with L1-penalty function. LASSO logistic regression was carried out by using glmnet R package, in which the optimal number of featured genes was determined when the lambda value was minimal. SVM-RFE machine learning algorithm was performed with five-fold cross-validation by using e1071 R package, in which the optimal number of featured genes was determined when the root mean square error (RMSE, cross-validation) was minimal. Afterwards, the overlapping featured genes were selected for further analysis. ROC curves and the values of area under the curve (AUC) were analyzed to assess the accuracy of the selected key genes obtained by the above algorithms.

### Construction of artificial neural network for discriminating PANoptosis related gene clusters

Based on the featured genes acquired above, random forest (RF) machine learning algorithm was used to further screen out featured genes via randomForest R package^53^. Firstly, the optimal number of the random forest trees was determined when the cross-validation error presented minimal. Then, the random forest with the optimal number of trees was constructed. In order to obtain featured genes with high importance, the feature importance for each gene was calculated and genes with feature importance > 10 were selected for further analysis.

Artificial neural network (ANN) exhibits powerful performance to clarify the association between complex and non-linear variables^54^ and was utilized as a special model to discriminate distinct PANoptosis related gene clusters in LGGs in our study. Firstly, a scoring system was constructed according to the expression levels of featured genes to eliminate the batch effect between the training dataset and validation datasets. For a featured gene that was upregulated in gene cluster A, the gene score was set to 1 when its expression level was higher than the median expression level and the gene score was set to 0 when its expression level was lower than the median level. For a featured gene that was downregulated in gene cluster A, the gene score was set to 1 when its expression level was lower than the median expression level and the gene score was set to 0 when its expression level was higher than the median level. Afterwards, the gene scores of the featured genes were treated as input values and the output layer was produced by connecting a hidden layer, in which the assigned weights were appropriately calculated. The number of neurons in the hidden layer was set to five. The number of neurons in the output layer was set to two, and the values of the two neurons in the output layer represented the possibility of LGG samples which were classified into gene clusters A or B.

### Validation of ANN in external datasets

One dataset (dataset ID: mRNAseq_325) obtained from CGGA database^55–58^ and one dataset (GSE43378) obtained from GEO database^59^ (Gene Expression Omnibus, https://www.ncbi.nlm.nih.gov/geo/) were treated as validation datasets, respectively. LGG patients in the validation datasets were classified into two PANoptosis related gene clusters based on the expression profiles of PANoptosis related gene signature by consensus clustering analysis. Similar gene scoring method was applied to three validation datasets and ANNs were constructed to verify the performance in discriminating the PANoptosis related gene clusters. Furthermore, we adopt the ten-fold cross-validation method to verify whether the model was overfitting. We randomly divided the training set into ten parts. Nine folds were used to train the model and make predictions on the remaining fold. This process is repeated 10 times until all samples have been validated once in the test set.

### Evaluation of the performance of ANN

Multiple metrics were calculated to better evaluate the performance of ANN in the training and validation datasets, including specificity, sensitivity, accuracy, and AUC^60^. In order to better characterize the evaluation metrics, LGGs in gene cluster A were defined as positive cases while LGGs in gene cluster B were defined as negative cases. The following values were used in the definition of the evaluation metrics: the number of true positives (TP), the number of false positives (FP), the number of true negatives (TN), and the number of false negatives (FN).

Specificity was defined as follows:

$$F3\,\boldsymbol{ }\boldsymbol{ }\boldsymbol{ }\boldsymbol{ }\boldsymbol{ }\boldsymbol{ }\boldsymbol{ }\boldsymbol{ }\boldsymbol{ }\boldsymbol{ }\boldsymbol{ }\mathrm{Specificity}=\frac{\mathrm{TN}}{\mathrm{TN}+\mathrm{FP}}.$$

Sensitivity was defined as follows:

$$F4\,\boldsymbol{ }\boldsymbol{ }\boldsymbol{ }\boldsymbol{ }\boldsymbol{ }\boldsymbol{ }\boldsymbol{ }\boldsymbol{ }\boldsymbol{ }\boldsymbol{ }\boldsymbol{ }\mathrm{Sensitivity}=\frac{\mathrm{TP}}{\mathrm{TP}+\mathrm{FN}}.$$

Accuracy was defined as follows:

$$F5\, \boldsymbol{ }\boldsymbol{ }\boldsymbol{ }\boldsymbol{ }\boldsymbol{ }\boldsymbol{ }\mathrm{accuracy}=\frac{\mathrm{TP}+\mathrm{TN}}{\mathrm{TP}+\mathrm{FP}+\mathrm{TN}+\mathrm{FN}}.$$

The AUC values ranging from 0 to 1, showed the capacity of ANN in distinguishing PANoptosis related gene clusters.

### Validation of the featured genes involved in ANN at protein level

Six genes including *GBP1, S100A4, TYMP, TNFRSF12A, VIM,* and *MSN* were randomly selected from the nine featured genes involved in ANN. The differential expression patterns of the above genes between normal and glioma tissues were identified on the Human Protein Atlas website (https://www.proteinatlas.org/)^61^.

Western blotting was implemented to further verify the differential expression levels of the above genes between normal and glioma tissues. Normal brain tissues were acquired from patients with epilepsy who received temporal lobe resection. Glioma tissues which were histologically diagnosed as grade II (G2), grade III (G3) and grade IV (G4) were obtained from patients who received tumor resection.

The collected tissues were separately homogenized and lysed in RIPA lysis buffer containing protease and phosphatase inhibitors at 0–4 °C. The homogenized protein samples were centrifuged at 1000*g* for 15 min at 4 °C to extract cytoplasmic proteins. The Bio-Rad protein assay kit was used to determine the protein concentration. The protein samples were homogenized with a prepared loading buffer and then boiled for 5 min at 100 °C. Equal amounts of protein samples were separated through SDS-PAGE at 80 V for 1 h. Afterwards, the protein samples were transferred onto polyvinylidene difluoride (PVDF) membranes at 50 V for 1 h. The membranes were incubated for 12 h with the following primary antibodies: GBP1, S100A4, thymidine phosphorylase (TYMP), TWEAKR (TNFRSF12A), vimentin (VIM), moesin (MSN), and β-actin. Subsequently, the membranes were incubated with secondary anti-rabbit or anti-mouse horseradish peroxidase (HRP) antibodies. Finally, the membranes were visualized with the enhanced chemiluminescence (ECL) solution.

### Statistical analysis

The prognosis of different subgroups was compared through the Kaplan–Meier survival analysis using the survminer and survival R packages. The log-rank test was used to perform statistical analysis. Comparisons between two groups were carried out by using the Wilcoxon rank-sum tests whereas comparisons among multiple groups were carried out using Kruskal–Wallis tests. Categorical variables between two groups were compared using the Chi-square tests. Continuous variables between two groups were compared with the independent student’s *t* test. Two-tailed p < 0.05 was considered statistically significant.

### Ethics approval

The study has been approved by the Ethics Committee of qingdao municipal hospital. We have obtained the approval and informed consent from the participates.

### Contribution to the field statement

PANoptosis, which shares common key features with pyroptosis, apoptosis and/or necroptosis, is determined as an inflammatory programed cell death (PCD) pathway and cannot be simply accounted for by any of these three identified PCD pathways alone. It has been recently reported that PANoptosis plays an important role in tumor progression. Scholars have revealed the inhibitory effect of PANoptosis on tumor growth in diverse cancer lineages, shedding more light on the investigation of biomarkers and therapeutic targets for patients. However, there are little studies focusing on the exploration of PANoptosis in gliomas. To date, there is no PANoptosis related gene signature has been identified with implications in prognosis in glioma patients. Due to highly variable prognosis in gliomas, it is becoming a hot spot to find biomarkers for predicting clinical outcomes. In our study, PANoptosis related gene clusters with distinct prognosis were identified based on the expression of PANoptosis related gene signature. Moreover, an ANN model based on machine learning methods was developed to characterize and discriminate distinct PANoptosis related gene clusters. Considering its prognostic values, PANoptosis related gene cluster can be an indicator for the heterogeneity of tumors across individuals and contributes to the development of personalized medicine, appealing implications in clinical management of glioma patients. The identification of PANoptosis related gene signature provided critical evidence for the exploration of PANoptosis in gliomas and sheds light on the investigation of promising therapeutics with respect to PANoptosis. Furthermore, given that the two PANoptosis related gene clusters represent distinct characteristics of tumor microenvironment (TME), this study facilitates in the investigation of immunotherapy in the future.

## Supplementary Information

Supplementary Figure 1.

Supplementary Figure 2.

Supplementary Figure 3.

Supplementary Figure 4.

Supplementary Figure 5.

Supplementary Figure 6.

Supplementary Table 1.

Supplementary Table 2.

Supplementary Table 3.

## Data Availability

We declare that the data sets supporting the findings of this study are available in the TCGA database (https://portal.gdc.cancer.gov/), CGGA database (http://cgga.org.cn/index.jsp), GEO database (https://www.ncbi.nlm.nih.gov/geo/) and Human Protein Atlas website (https://www.proteinatlas.org/). Glioma samples for western blotting were obtained from the department of neurosurgery of qingdao municipal hospital. We confirm that all methods were performed in accordance with the relevant guidelines and regulations.
