# Supplementary material for: Construction of a machine learning-based artificial neural network for discriminating PANoptosis related subgroups to predict prognosis in low-grade gliomas
Source: Sci Rep. 2022 Dec 21;12:22119. doi: 10.1038/s41598-022-26389-3 (PMC9770564; doi:10.1038/s41598-022-26389-3)
Supplement: Supplementary file 1 — Supplementary Figure 1. [file 41598_2022_26389_MOESM1_ESM.pdf]

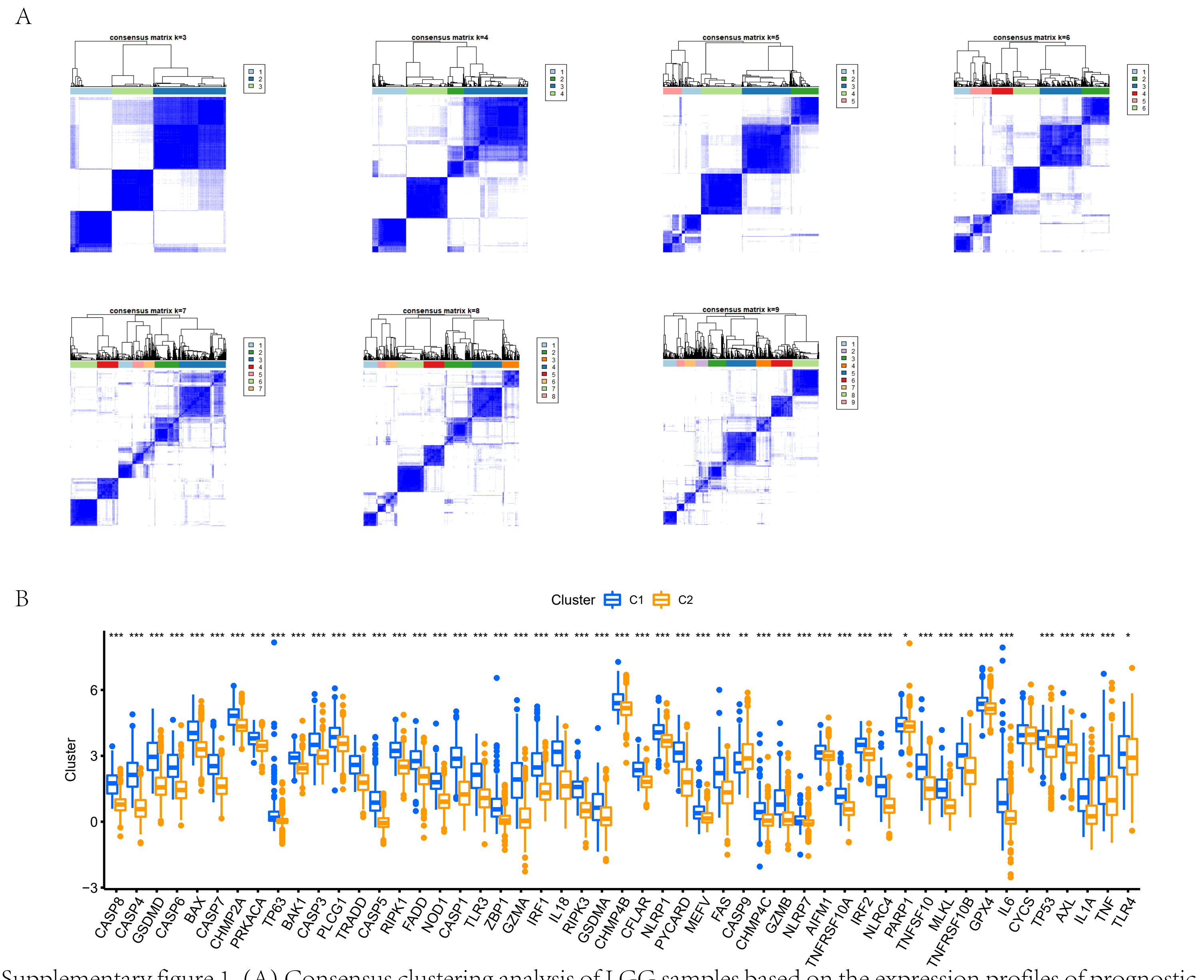

Supplementary figure 1. (A) Consensus clustering analysis of LGG samples based on the expression profiles of prognostic PRGs when k ranged from 3 to 9. (B) Comparisons of the expression levels of prognostic PRGs between two PANoptosis related molecular patterns. LGG, low-grade glioma; PRG, PANoptosis related gene. * $p < 0.05$, ** $p < 0.01$, *** $p < 0.001$.
