# Supplementary material for: Construction of a machine learning-based artificial neural network for discriminating PANoptosis related subgroups to predict prognosis in low-grade gliomas
Source: Sci Rep. 2022 Dec 21;12:22119. doi: 10.1038/s41598-022-26389-3 (PMC9770564; doi:10.1038/s41598-022-26389-3)
Supplement: Supplementary file 3 — Supplementary Figure 3. [file 41598_2022_26389_MOESM3_ESM.pdf]

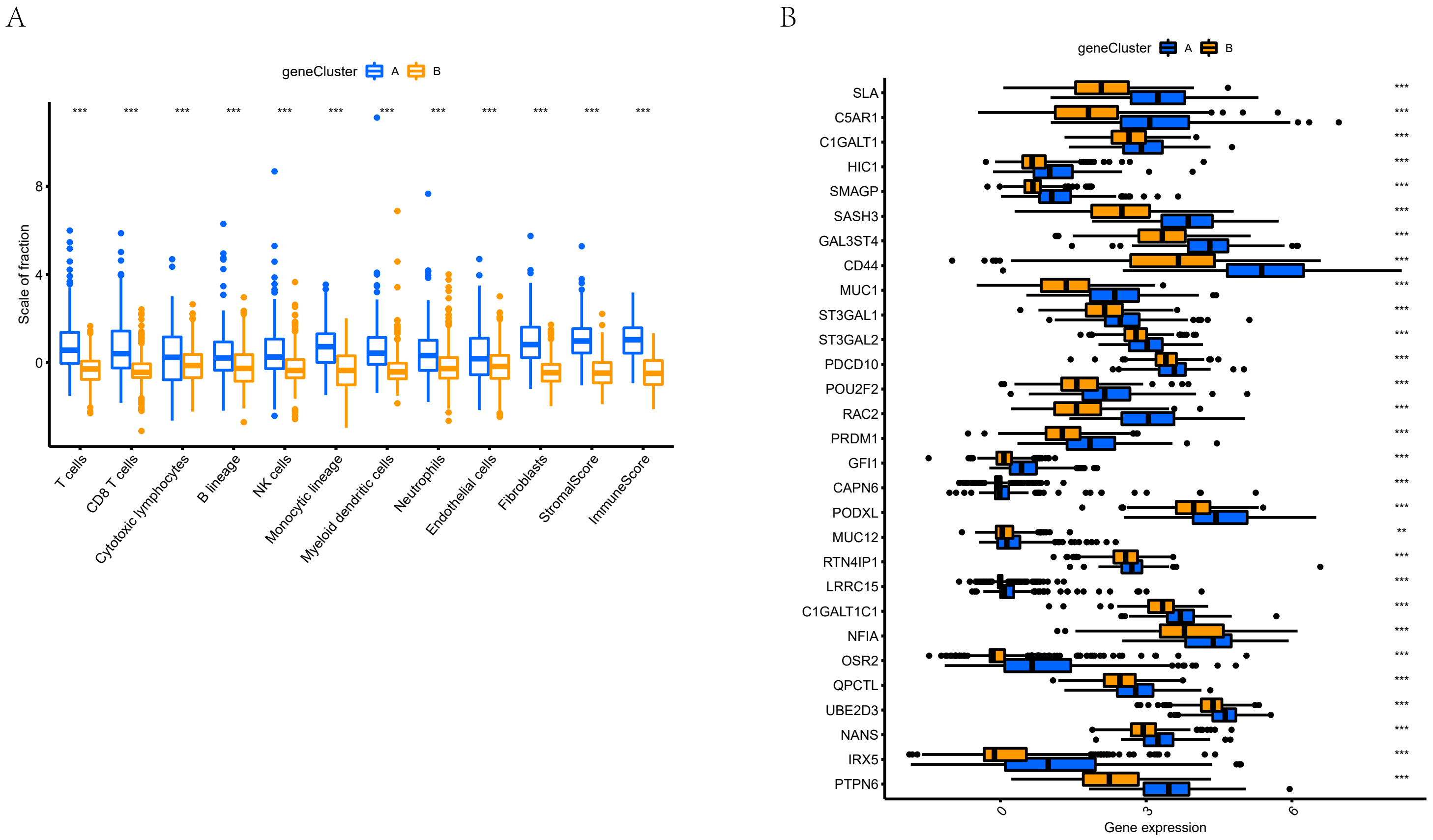

Supplementary figure 3. (A) Comparisons of the abundance of infiltrating cells in TME between PANoptosis related gene clusters. (B) Comparisons of the expression levels of genes involved in negative regulation of ADCP. TME, tumor microenvironment; ADCP, antibody-dependent cellular phagocytosis; * $p < 0.05$, ** $p < 0.01$, *** $p < 0.001$.
