# Supplementary material for: Construction of a machine learning-based artificial neural network for discriminating PANoptosis related subgroups to predict prognosis in low-grade gliomas
Source: Sci Rep. 2022 Dec 21;12:22119. doi: 10.1038/s41598-022-26389-3 (PMC9770564; doi:10.1038/s41598-022-26389-3)
Supplement: Supplementary file 4 — Supplementary Figure 4. [file 41598_2022_26389_MOESM4_ESM.pdf]

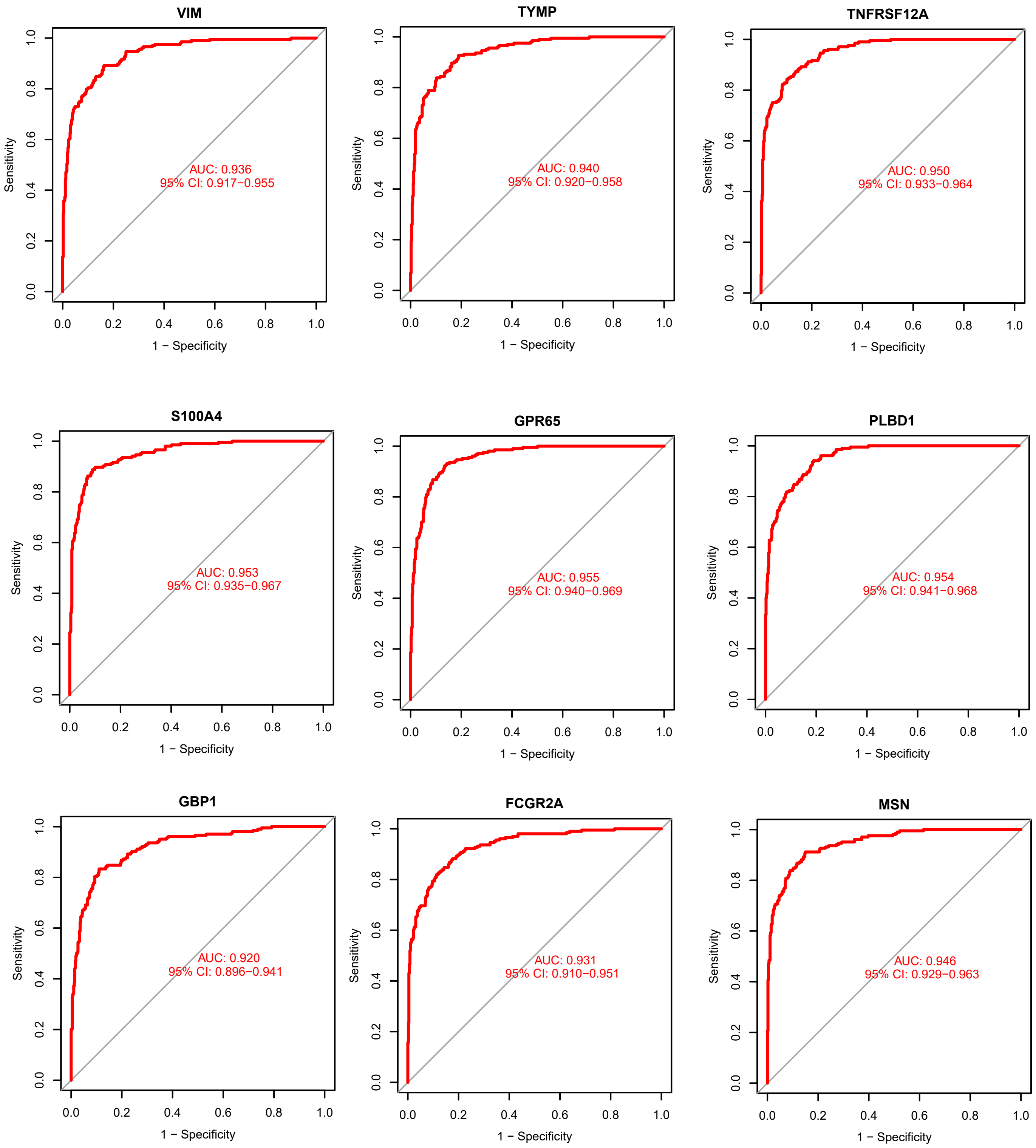

Supplementary figure 4. ROC curves demonstrating the efficacy of the nine featured genes in discriminating two PANoptosis related gene clusters. ROC, receiver operating characteristic
