# Supplementary material for: Construction of a machine learning-based artificial neural network for discriminating PANoptosis related subgroups to predict prognosis in low-grade gliomas
Source: Sci Rep. 2022 Dec 21;12:22119. doi: 10.1038/s41598-022-26389-3 (PMC9770564; doi:10.1038/s41598-022-26389-3)
Supplement: Supplementary file 5 — Supplementary Figure 5. [file 41598_2022_26389_MOESM5_ESM.pdf]

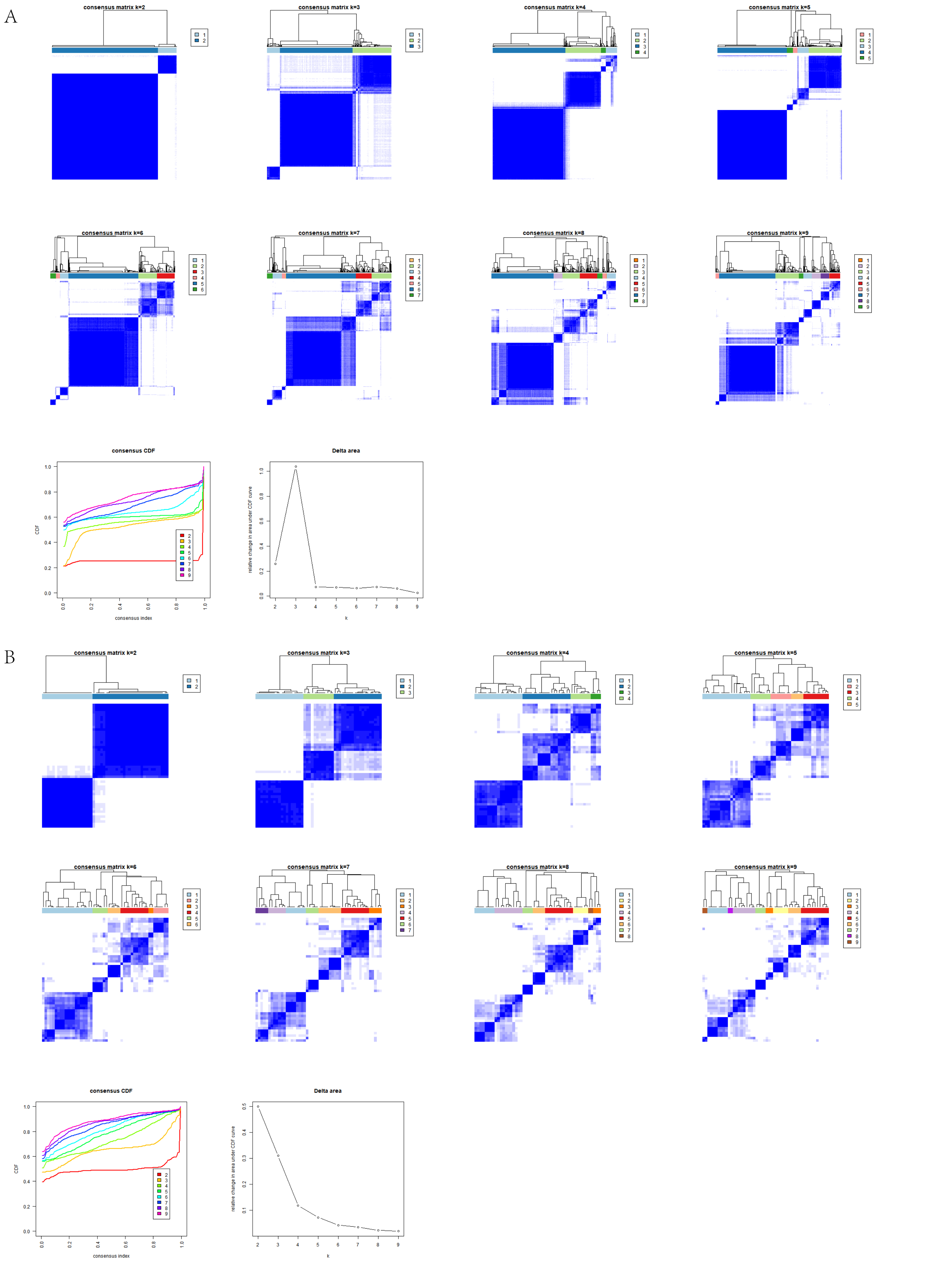

Supplementary figure 5. (A) Consensus clustering analysis of glioma samples based on PANoptosis related gene signature in validation data set from CGGA database (data set ID: mRNAseq_325). (B) Consensus clustering analysis of glioma samples based on PANoptosis related gene signature in validation data set from GEO database (data set ID: GSE43378).
