# Supplementary material for: Construction of a machine learning-based artificial neural network for discriminating PANoptosis related subgroups to predict prognosis in low-grade gliomas
Source: Sci Rep. 2022 Dec 21;12:22119. doi: 10.1038/s41598-022-26389-3 (PMC9770564; doi:10.1038/s41598-022-26389-3)
Supplement: Supplementary file 6 — Supplementary Figure 6. [file 41598_2022_26389_MOESM6_ESM.pdf]

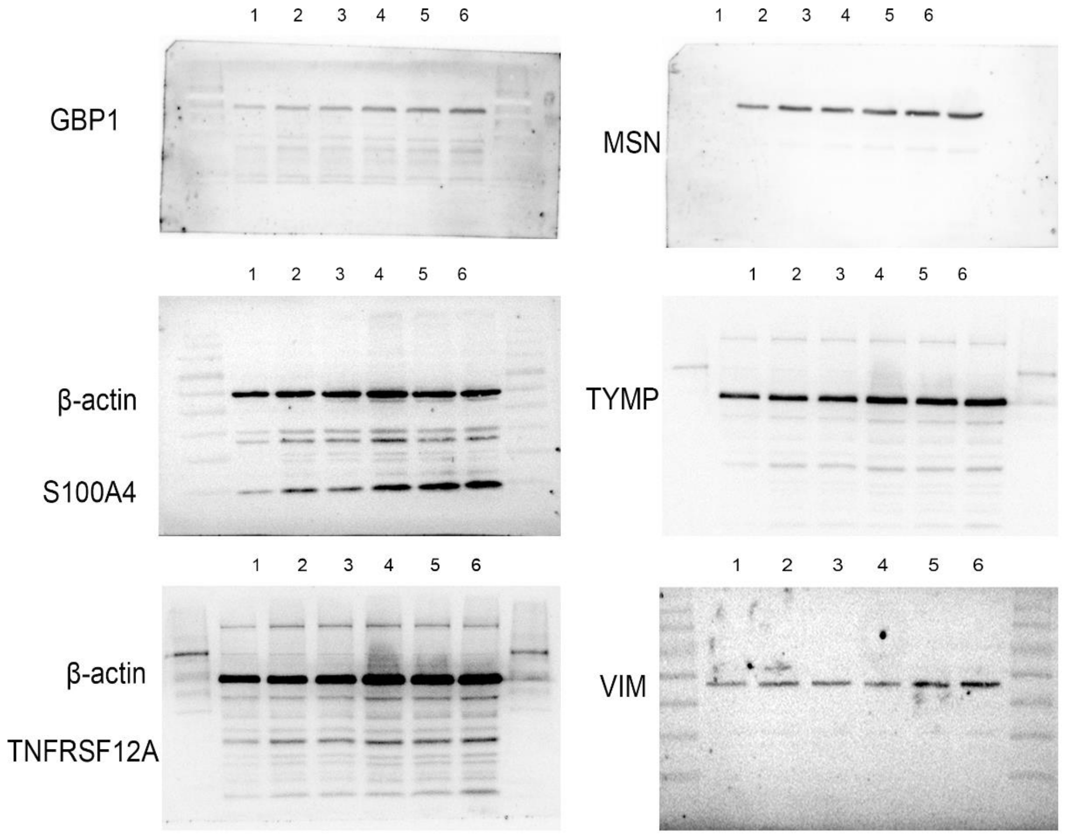

Supplementary figure 6 Identification of the featured genes by western blotting, in which lane 1 represented normal brain tissues, lane 2 and 3 represented grade II glioma tissues, lane 4 and 5 represented grade III glioma tissues, lane 6 represented grade IV glioma tissues. Control: normal brain tissue.
