# Supplementary material for: Construction of a machine learning-based artificial neural network for discriminating PANoptosis related subgroups to predict prognosis in low-grade gliomas
Source: Sci Rep. 2022 Dec 21;12:22119. doi: 10.1038/s41598-022-26389-3 (PMC9770564; doi:10.1038/s41598-022-26389-3)
Supplement: Supplementary file 7 — Supplementary Table 1. [file 41598_2022_26389_MOESM7_ESM.docx]

**Supplementary table 1. Clinicopathological features of LGG patients in TCGA database**

| Covariates |  | Total | geneCluster A | geneCluster B |
| --- | --- | --- | --- | --- |
| Age | <45 | 301(59.02%) | 69(46.62%) | 232(64.09%) |
|  | >=45 | 209(40.98%) | 79(53.38%) | 130(35.91%) |
| Grade | G2 | 248(48.72%) | 35(23.65%) | 213(59%) |
|  | G3 | 261(51.28%) | 113(76.35%) | 148(41%) |
| Histologic  Type | Astrocytoma | 192(37.65%) | 94(63.51%) | 98(27.07%) |
|  | Oligoastrocytoma | 128(25.1%) | 29(19.59%) | 99(27.35%) |
|  | Oligodendroglioma | 190(37.25%) | 25(16.89%) | 165(45.58%) |
| IDH1  Mutation | Mutant | 91(72.8%) | 9(30%) | 82(86.32%) |
|  | Wildtype | 34(27.2%) | 21(70%) | 13(13.68%) |
| Gender | Female | 228(44.71%) | 64(43.24%) | 164(45.3%) |
|  | Male | 282(55.29%) | 84(56.76%) | 198(54.7%) |
| Therapy  Outcome | Complete Remission/Response | 84(36.21%) | 19(30.16%) | 65(38.46%) |
|  | Partial Remission/Response | 50(21.55%) | 6(9.52%) | 44(26.04%) |
|  | Progressive Disease | 39(16.81%) | 21(33.33%) | 18(10.65%) |
|  | Stable Disease | 59(25.43%) | 17(26.98%) | 42(24.85%) |
