# Supplementary material for: Construction of a machine learning-based artificial neural network for discriminating PANoptosis related subgroups to predict prognosis in low-grade gliomas
Source: Sci Rep. 2022 Dec 21;12:22119. doi: 10.1038/s41598-022-26389-3 (PMC9770564; doi:10.1038/s41598-022-26389-3)
Supplement: Supplementary file 8 — Supplementary Table 2. [file 41598_2022_26389_MOESM8_ESM.docx]

**Supplementary table 2. Clinicopathological features of LGG patients in CGGA database (DataSet ID: mRNA-array_301)**

| Covariates |  | Total | geneCluster A | geneCluster B |
| --- | --- | --- | --- | --- |
| TCGA_subtypes | Classical | 7(4.02%) | 6(10.71%) | 1(0.85%) |
|  | Mesenchymal | 33(18.97%) | 33(58.93%) | 0(0%) |
|  | Neural | 69(39.66%) | 9(16.07%) | 60(50.85%) |
|  | Proneural | 65(37.36%) | 8(14.29%) | 57(48.31%) |
| Recurrent_type | Primary | 156(89.66%) | 44(78.57%) | 112(94.92%) |
|  | Recurrent | 18(10.34%) | 12(21.43%) | 6(5.08%) |
| Grade | G2 | 117(67.24%) | 26(46.43%) | 91(77.12%) |
|  | G3 | 57(32.76%) | 30(53.57%) | 27(22.88%) |
| Gender | Female | 73(41.95%) | 18(32.14%) | 55(46.61%) |
|  | Male | 101(58.05%) | 38(67.86%) | 63(53.39%) |
| Age | <45 | 121(70.35%) | 35(63.64%) | 86(73.5%) |
|  | >=45 | 51(29.65%) | 20(36.36%) | 31(26.5%) |
| Radio_status | treated | 139(83.23%) | 44(81.48%) | 95(84.07%) |
|  | untreated | 28(16.77%) | 10(18.52%) | 18(15.93%) |
| Chemo_status | treated | 69(43.95%) | 29(53.7%) | 40(38.83%) |
|  | untreated | 88(56.05%) | 25(46.3%) | 63(61.17%) |
| IDH_mutation  status | Mutant | 112(65.12%) | 28(50.91%) | 84(71.79%) |
|  | Wildtype | 60(34.88%) | 27(49.09%) | 33(28.21%) |
| 1p19q_Codeletion  status | Codel | 16(30.19%) | 2(11.76%) | 14(38.89%) |
|  | Non-codel | 37(69.81%) | 15(88.24%) | 22(61.11%) |
| MGMTp_methylation  status | methylated | 46(28.75%) | 16(31.37%) | 30(27.52%) |
|  | un-methylated | 114(71.25%) | 35(68.63%) | 79(72.48%) |
