# Supplementary material for: Construction of a machine learning-based artificial neural network for discriminating PANoptosis related subgroups to predict prognosis in low-grade gliomas
Source: Sci Rep. 2022 Dec 21;12:22119. doi: 10.1038/s41598-022-26389-3 (PMC9770564; doi:10.1038/s41598-022-26389-3)
Supplement: Supplementary file 9 — Supplementary Table 3. [file 41598_2022_26389_MOESM9_ESM.docx]

| **TN** | **FN** | **FP** | **TP** | **Specificity** | **Sensitivity** | **Accuracy** | **AUC** |
| --- | --- | --- | --- | --- | --- | --- | --- |
| 108 | 3 | 11 | 50 | 0.908 | 0.943 | 0.919 | 0.9685455 |
| 109 | 7 | 3 | 53 | 0.973 | 0.883 | 0.942 | 0.9661069 |
| 119 | 8 | 8 | 37 | 0.937 | 0.822 | 0.907 | 0.9649785 |
| 111 | 6 | 11 | 44 | 0.91 | 0.88 | 0.901 | 0.9518548 |
| 121 | 8 | 2 | 41 | 0.984 | 0.837 | 0.942 | 0.9705859 |
| 117 | 11 | 9 | 35 | 0.929 | 0.761 | 0.884 | 0.9549875 |
| 113 | 1 | 10 | 48 | 0.919 | 0.98 | 0.936 | 0.9524882 |
| 106 | 2 | 8 | 56 | 0.93 | 0.966 | 0.942 | 0.9955181 |
| 116 | 4 | 6 | 46 | 0.951 | 0.92 | 0.942 | 0.9639397 |
| 114 | 5 | 10 | 43 | 0.919 | 0.896 | 0.913 | 0.9692007 |

**supplementary table3 :accuracy of the ANN model in the 10-fold cross-validation**
